# Monumental Physicians

**Published:** 1852-03

**Authors:** 


### EDITORIAL DEPARTMENT.

---

*Monumental Physicians.*—"It takes all sorts of people to make a world," is a homely apothegm, containing a verity indisputable. So every calling in life is made up of members exhibiting diversities of character. That of Medicine, as well as other pursuits, furnishes exemplifications of this truism. For critical examination we may subdivide the professors of the healing art into various groups, each distinguished by characteristic traits from the others. An attempt at a classification of this sort may have some interest, and possibly be attended with profit. The disposition to criticise others is a common weakness, not always altogether amiable. It is often easier to do this than to recognize one's own peculiarities. That the faults of our brethren are viewed *microscopically*, while the personal defects of the observer never enter the field of his own vision, is but too frequently true. We cannot see ourselves as others see us. Nevertheless, if conducted with a right spirit, it may not be wholly a bootless occupation to study human character *objectively* as a matter of curious research; and such a study, indeed, may be useful, if the critic do not omit *subjective* examinations, and if he endeavor to apply the results of his inquiries, in either direction, to his own improvement.

The idea has occurred to us to portray some of the different classes of physicians, and by way of breaking ground in this unoccupied field of exploration, we propose, at this time, to say a few words of a group which we will designate the *monumental.* The reader might perhaps be somewhat nonplussed in trying to divine the significance of this adjective *monumental* as applied to a portion of the medical profession. The term, however, is something more than a mere rhetorical conceit. An eloquent teacher, in his parting remarks to a class of medical students, enjoined upon them the importance of unceasing application if they did not wish to remain living monuments of what medical science had been, instead of exemplifying its actual condition. This happy thought has suggested the epithet. We observe everywhere in the ranks of the profession, a certain number who represent the past, rather than the present. They are living monuments of former stages of the career of science. To understand their position our examination must be retrospective. They do not reflect science as it is, still less as it is to be, but perpetuate it as it has been.

We have said that monumental physicians exist every where. There are none of our readers who cannot indicate, in their respective circles, persons of this stamp. They have, moreover, always existed. They are not of modern origin. They were abundant at the time of Harvey, if there be truth in the often reiterated statement that none of his brethren who were over forty years of age ever admitted the verity of the discovery of the circulation. These monumental physicians doubtless continued to represent in their teachings and practice the old doctrine that the arteries were air tubes! Harvey himself had something of the monumental element in his composition, for he obstinately refused to admit the discovery of the absorbent vessels. Jenner was surrounded with monumental physicians who ridiculed, scouted, and opposed a discovery which has saved millions of human beings from a loathsome disease. A marble monument, at this late day, is to be reared to the memory of that great benefactor of the race. One cannot help thinking how much better than this had it been if his monumental contemporaries had not withheld the encouragement and meed of praise which were his due.

Monumental physicians of the present time do not all represent the same period of the past. A few are the living representatives of medicine as it was forty or fifty years ago. Some carry us back a score of years; and others are only removed a single decade. Of the monuments of the first class in the order of time, we find some adhering still to the teachings of the immortal Rush. Such an one believes in the unity of disease, and, as a pr c-titioner, his tendencies are sanguinary. His lancet is bright and sharp, and always on hand for ready use. Bloodletting, so potent either for good or ill, is a daily operation with him. He is sure not to run any risk of letting his patients die for the want of this remedy. Other monuments of the same chronological class, (but these are comparatively few in number) perpetua the views and practice of the gifted, eccentric Brown. Thus, living monuments represent not only the different stadia of scientific progress, but different ideas incident to the state of science at the same era.

In monuments of a more modern date, Broussais still lives, and flourishe with perennial vigor. Inflammation the fundamental pathological element in all diseases, and, deductively, leeches, with gum water for diet, form the basis of practice. The Hepatic pathology has its numerous monumental representatives. Regarded in the light of this pathology, a portion of the primeval curse was the infliction of a liver; the bile, as if it were a part of the innate depravity of man, is to be ejected by emetics and cathartics, and so long as a drop remains, the physical man is unregenerate.

To describe the different kinds of monumental representatives would involve a catalogue of all the more salient points of the history of medicine within the memory of those now living. We have not the materials for an analysis of this description. Our object is only to point out the distinctive characters which belong to the class. The portrait of each specimen would present a certain individuality of physiognomy. The study, in its details, can be pursued by each observer with the models before him among those which fall within the scope of his personal acquaintance. Daguerreotype likenesses might be furnished in abundance, but the end would hardly repay the trouble.

Collectively, the members of the group under consideration have certain features in common which distinguish the genus, without descending to the multiplicity of particulars which pertain to individuals. The leading, most distinctive quality, is *fixedness of position.* While the progress of science and art is onward, they are stationary, occupying the same spot, with eyes reverted, not remembering Lot's wife! They can perceive nothing encouraging or attractive in recent developments, and still less is there aught in the prospective to awaken enthusiasm. Your monumental physicians are fond of talking of the superiority of times gone by. They are the very antipodes of the optimist. 'Medicine has lost much of its ancient character and claims' is a favorite remark. 'The profession is deteriorating.' 'There is no telling what is to be the end.' They even entertain serious apprehensions lest, with the march of pseudo-civilization, the whole world will by and by go over to quackery! 'The various forms of empiricism were certainly not so rampant in their young days.' They have a very low opinion of the younger class of physicians. They regard with distrust organizations for mutual improvement and the advancement of science. Consistency with them is indeed a jewel, for they never change. Experience is, in their view, the only source of scientific illumination, and the more experience they get the more is their own confidence in their long established views and practice sure to be enhanced. They are outraged at the audacity of a newer generation in thinking and acting for themselves. Seniority, in their estimation, claims more than respect, it should be invested with authority, forgetting that it is as ungraceful on the one hand to insist upon the homage due to age, as it is, on the other hand, to withhold it in proper measure. But we need not extend the list of generic attributes; what has been mentioned will suffice for all purposes of *differential diagnosis.*

Let it not be said that the tenor of these remarks is calculated to depreciate the past condition of medicine. In placing a just regard on the character of our science at any former epoch, we are not alone to compare it with its present position. It would be indeed humiliating if such a comparison did not exhibit a contrast more or less striking. Constant progress is the law of this, as of other departments of knowledge which do not spring from revelation. It is by the labors of its truth-loving votaries (not living monuments) in every age, that Medicine has advanced thus far in its onward course. It has become what it is by the aggregation of the products of each successive generation. Does any one doubt its progressive character? Such a skeptic is too ignorant of the subject to be qualified to form an opinion. Mark the changes that have transpired during the last quarter of a century! Look at the results of the application of physical signs to the diagnosis of the diseases of the heart and lungs; and, more recently, of the introduction of the microscope into the study of the solids and fluids in health and disease! Look at the nosological space now occupied by the neuroses, and consider that twenty years ago the misnomer *tic doloreux* was almost the sole representative of neuralgic affections. Look at the influence on practice which has resulted from a better appreciation of anæmia, from the recognition of the self-limited duration of various diseases, from the study of the natural history of essential fevers, from more correct views of the pathology of tuberculosis, from the light shed on the relations of the different alimentary constituents to nutrition and animal temperature, etc. etc. etc. Some diseases, in this short period, have taken entirely new situations, e. g. diabetes has become an affection of assimilation, not of secretion; and, again, new diseases have been discovered, e. g. the *morbus Brightii.* All this is, we trust, supererogatory for our readers. But, we repeat, to place a just estimate upon any period in the past, we are to exhibit it not only in contrast with the present, but with a period still more remote. Medicine, a quarter of a century ago, was far in advance of the position which it held at the remove of a half century. Is it any disparagement of the physical sciences of by-gone years that such rapid strides have lately been made in their applications to useful arts? Is the brilliancy of the discovery of Franklin dimmed by the fact that the electrical fluid has just been made subservient to telegraphic purposes? It was not less a glorious achievement, not very many years since, for Fulton to have performed the voyage from Albany to New York in a vessel propelled by steam, because at a still later period the more gigantic enterprise of crossing the Atlantic by means of the same motive power has been accomplished. Let us honor the past, and reverence the names of those to whose genius and industry the world is indebted for its present stock of knowledge. But let the records of history and a just sense of our obligations take the place of living monuments, at least in medical science.

Will the captious reader say that our remarks are calculated to disparage a portion of the medical profession. The only rejoinder to this criticism is an appeal to the justness of the remarks. Are there not physicians even at the present moment who continue to sneer at the stethoscope as a useless toy? Are there not still more who virtually repudiate physical diagnosis? And is there not room for a similar question with respect to almost every other development of modern science?

A homily is not complete without an exhortation. The Science of Medicine is emphatically progressive. Not only has it advanced up to the present time, but its course is still to be onward. Do we represent its present or past condition? This is an inquiry for individual application. But this is only one of the points of view in which the subject comes home to our reflections. Shall we keep pace with its progressive march, or shall we at a future date, if life be spared, be the representatives of what it now is, and not of what it is then to be? It is only by continued exertions, guided by a proper recognition of the law of progress, that we shall avoid becoming, if we are not already, *monumental physicians.*

